# Cloning and Functional Identification of Phosphoethanolamine Methyltransferase in Soybean (*Glycine max*)

**DOI:** 10.3389/fpls.2021.612158

**Published:** 2021-07-27

**Authors:** Xiaomin Ji, Xiaoyue Wu, Wei Chen, Qianhui Yuan, Yixin Shen, Yingjun Chi

**Affiliations:** College of Agro-Grassland Science, Nanjing Agricultural University, Nanjing, China

**Keywords:** phosphoethanolamine methyltransferase, phosphatidylcholine, lecithin, *Glycine max*, phospholipid metabolism

## Abstract

Phosphoethanolamine methyltransferase (PEAMT), a kind of S-adenosylmethionine-dependent methyltransferases, plays an essential role in many biological processes of plants, such as cell metabolism, stress response, and signal transduction. It is the key rate-limiting enzyme that catalyzes the three-step methylation of ethanolamine-phosphate (P-EA) to phosphocholine (P-Cho). To understand the unique function of PEAMT in soybean (*Glycine max*) lipid synthesis, we cloned two phosphoethanolamine methyltransferase genes *GmPEAMT1* and *GmPEAMT2*, and performed functional identification. Both GmPEAMT1 and GmPEAMT2 contain two methyltransferase domains. GmPEAMT1 has the closest relationship with MtPEAMT2, and GmPEAMT2 has the closest relationship with CcPEAMT. GmPEAMT1 and GmPEAMT2 are located in the nucleus and endoplasmic reticulum. There are many light response elements and plant hormone response elements in the promoters of *GmPEAMT1* and *GmPEAMT2*, indicating that they may be involved in plant stress response. The yeast *cho2 opi3* mutant, co-expressing *Arabidopsis thaliana* phospholipid methyltransferase (PLMT) and GmPEAMT1 or GmPEAMT2, can restore normal growth, indicating that GmPEAMTs can catalyze the methylation of phosphoethanolamine to phosphate monomethylethanolamine. The heterologous expression of *GmPEAMT1* and *GmPEAMT2* can partially restore the short root phenotype of the *Arabidopsis thaliana peamt1* mutant, suggesting GmPEAMTs have similar but different functions to AtPEAMT1.

## Introduction

Phosphatidylcholine (PC) is a type of cell membrane phospholipid (Bolognese and McGraw, 2000), which plays a vital role in various eukaryotic organisms. PC is necessary for the rapid proliferation of malaria parasites in human red blood cells, so its synthesis pathway has become a potent target for malaria chemotherapy (Pessi et al., 2004). PC can also be used as a special nutritional additive to supplement the phospholipids needed in the animals to improve the immunity and survival rate of pups (Zhang et al., 2006). As an essential membrane structure lipid, PC can repair biological membrane damage caused by free radical production and improve antioxidant activity in plants (Asano et al., 2015). PC can be hydrolyzed to produce phosphatidic acid (PA) and choline (Cho) by phosphatase D (Bolognese and McGraw, 2000). As a second messenger in plant cells, PA plays an important role in the signal transduction pathways related to plant stress response (Hong et al., 2010). In spinach (*Spinacia oleracea* L.), sugar beet (*Beta vulgaris* L.), barley (*Hordeum vulgare* L.), wheat (*Triticum aestivum* L.), rye (*Secale cereale* L.), and other plants, PC is the synthetic precursor of glycine betaine (GB) (McNeil et al., 2001). GB is a kind of osmotic protection agent that exists universally in plants. It can increase the osmotic pressure in the cytoplasm, stabilize protein complexes, protect plant cell membranes under stress conditions, and maintain their lipid membrane integrity (Sakamoto and Murata, 2000; Sakamoto et al., 2000).

In mammals and fungi, there are two main pathways for the synthesis of PC: (1) The nucleotide pathway, also known as the “Kennedy pathway” (Kennedy and Lehninger, 1949) or the cytidine diphosphate-choline (CDP-Cho) pathway, which is highly conserved in eukaryotes. Free choline is phosphorylated to form P-Cho by choline kinase, and then P-Cho is converted into CDP-Cho through cytidine triphosphate: phosphocholine cytidylyl transferase (CTP). Then it is converted into PC by cytidine diphosphate-choline: 1,2-diacylglycerol choline phosphotransferase (CEPT). (2) The methylation pathway, with S-adenosylmethionine (SAM) as the methyl donor, phosphatidylethanolamine (Ptd-EA) is converted to PC through three-step continuous methylation catalyzed by phospholipid methyltransferase (PLMT) (Carman and Henry, 1989).

The synthesis pathway of PC in plants is relatively complex, requiring the joint contribution of the nucleotide pathway and the methylation pathway (Hitz et al., 1981). Similar to mammals and yeast, free choline can also be finally converted into PC through the nucleotide pathway in plants. However, when there is no free choline in plants, or when the Kennedy pathway is blocked, the methylation of phosphoethanolamine (ethanolamine-phosphate, P-EA) acts as the first step in the process of PC biosynthesis. Under the catalysis of plant-specific phosphoethanolamine methyltransferase (PEAMT), phosphoethanolamine (ethanolamine-phosphate, P-EA) is converted into phosphorylcholine (P-Cho) *via* two intermediate products, monomethylethanolamine-phosphate (P-MMEA) and dimethylethanolamine-phosphate (P-DMEA). And, then, it merges into the nucleotide pathway to form PC finally. Alternatively, P-MMEA and P-DMEA can also produce phosphatidylmonomethylethanolamine (Ptd-MMEA) and phosphatidyldimethylethanolamine (Ptd-DMEA), respectively, through the nucleotide pathway, and then PC can be produced by PLMT at the phosphatidyl level (Lykidis, 2007). It is worth noting that PLMT that has been discovered in plants so far can only use Ptd-MMEA or Ptd-DMEA as a catalytic substrate (Keogh et al., 2009). Therefore, even though there are considerable differences in phosphate-level methylation and phosphatidyl-level methylation among different plants, the methylation of P-EA catalyzed by PEAMT to produce P-MMEA is the only entrance for PC synthesis (Chen et al., 2018a). Given the critical role of PEAMT in the PC synthesis, it has been studied in plants such as *Arabidopsis thaliana* (Bolognese and McGraw, 2000), wheat (Charron et al., 2002), and corn (*Zea mays* L.) (Wu et al., 2007).

Phosphatidylcholine is essential to improve the quality of soybean (*Glycine max*), which is an important source of plant protein and edible oil. In addition, soybean lecithin, mainly composed of PC, has been widely used as a natural raw material in the food, medical, and cosmetics industries (Gu et al., 2001). The PC synthesis pathway of soybean is slightly different from other plants, mainly in the methylation reactions at the phosphate level (Datko and Mudd, 1988a), suggesting a special function of GmPEAMTs. Since PEAMTs have not been identified from soybean or other legumes, we isolated the coding sequences and promoters of *PEAMTs* from soybean and conducted a systematic study on them. The findings of this study make us better understand the unique role of *GmPEAMTs* in the synthesis of PC. It is also of guiding significance for promoting the molecular breeding process of legumes.

## Materials and Methods

### Plant Materials and Growth Conditions

*Arabidopsis thaliana* T-DNA mutant *peamt1* (SALK_036291) was obtained from ABRC^1^. *Arabidopsis thaliana* ecotype Col-0 and *peamt1* mutant were grown under 16-h light/8-h dark conditions at 24°C/20°C. A ½-MS solid medium was used for plant culture. *Glycine max* (cv. “Williams82”) was cultivated in soil at 24°C with 12-h light/12-h dark.

### Identification of *GmPEAMT* and Its Promoter

Potential *GmPEAMT* sequences of soybean were identified, using BLASTN at the Phytozome v12.1 website^2^ based on *Arabidopsis thaliana PEAMT1* (*At3g18000*). The results were filtered with a score value ≥100 and an *E*-value ≤ 1e-10. The filtered genes were further analyzed for the potential domains, using SMART^3^ (Letunic and Bork, 2018). The promoter region and the coding sequences of *GmPEAMT* were amplified by PCR (primers are listed in Supplementary Table 1), cloned into the pMD^TM^19-T vector (TaKaRa, Japan) and sequenced (Tsingke, China).

### Sequence and Phylogenetic Analysis

Genome structure was analyzed by online program GSDS^4^. Structural domains were predicted through SMART. The sequences of other plant PEAMTs were obtained from NCBI, using BLASTP. Multiple alignments were conducted with ClustalX 2.0 and viewed with GeneDOC. The transmembrane domains were predicted by TMHMM^5^. Phylogenetic analyses were conducted in MEGA X. The evolutionary history was inferred, using the neighbor-joining method. The bootstrap set value was 1,000 replicates. The possible *cis-*acting elements in the promoters were predicted by PlantCARE (Lescot et al., 2002) and visualized with TBtools (Chen et al., 2020).

### RNA Extraction and RT-qPCR

Total RNA was isolated from soybean and reverse transcribed by the HiScript^®^ II Q RT SuperMix Kit (Vazyme, China). Tissue expression was determined by qRT-PCR, using QuantStudio 5 (Applied Biosystems, United States), with AceQ^®^ qPCR SYBR^®^ Green Master Mix (Vazyme, China). A relative transcript level was calculated by the method of 2^–ΔΔCt^ (Livak and Schmittgen, 2001) and normalized to the *GmTUBLIN* (*NM_001252709.2*) transcript level. The primers are listed in Supplementary Table 1.

### Subcellular Localization of GmPEAMT

The CDS of *GmPEAMT1* and *GmPEAMT2* were inserted into the vector pFGC5941-GFP to generate GmPEAMT: GFP fusion protein (primers are listed in Supplementary Table 1). By infiltrating with *Agrobacterium tumefaciens* (GV3101), containing the recombinant plasmids, *GmPEAMT1* and *GmPEAMT2* were transiently expressed in *Nicotiana benthamiana* leaves. The fluorescence signals were detected by a confocal laser microscope LSM510 (Zeiss, Germany) 2 days after infiltration.

### Transgenic Yeasts

*GmPEAMT1*, *GmPEAMT2*, and Arabidopsis *PEAMT1* were amplified from the cDNA (primers are listed in Supplementary Table 1) and inserted into the vector pESC-Leu through homologous recombination. Arabidopsis *PLMT* (*AT1G80860*) was amplified and linked to the vector pYES2. These constructs, as well as empty vectors pESC-Leu and pYES2, were transformed into *Saccharomyces cerevisiae* mutant *cho2 opi3* (ScCHO33), respectively (Pessi et al., 2005).

For spotting assays, single clones for each type of transformed yeast were firstly cultivated on liquid SD-leucine-uracil media, supplemented with 1-mM choline and 2% (w/v) glucose at 30°C, 200 rpm agitation until OD_600_ reached about 1. Yeast cells were harvested by centrifugation at 4,000 *g* for 5 min and then washed two times with ddH_2_O. OD_600_ was adjusted to 0.1 with ddH_2_O. About 10-μl yeast cells were then spotted on agar, supplemented with minimal media and 2% (w/v) galactose, and 1-mM ethanolamine or 1-mM monomethylethanolamine or 1-mM choline. Plates were incubated at 37°C for 2–5 days (Chen et al., 2018a).

### Transgenic Plants

The coding region of *GmPEAMT1* and *GmPEAMT2* was amplified and recombined into the vector pFGC5941, which was then transferred into the *peamt1* background by the *Agrobacterium tumefaciens*-mediated floral dip method (Davis et al., 2009; primers are listed in Supplementary Table 1). Transformants were screened on ½-MS media, containing Basta.

## Results

### Identification of *GmPEAMT1* and *GmPEAMT2*

**FIGURE 1:**
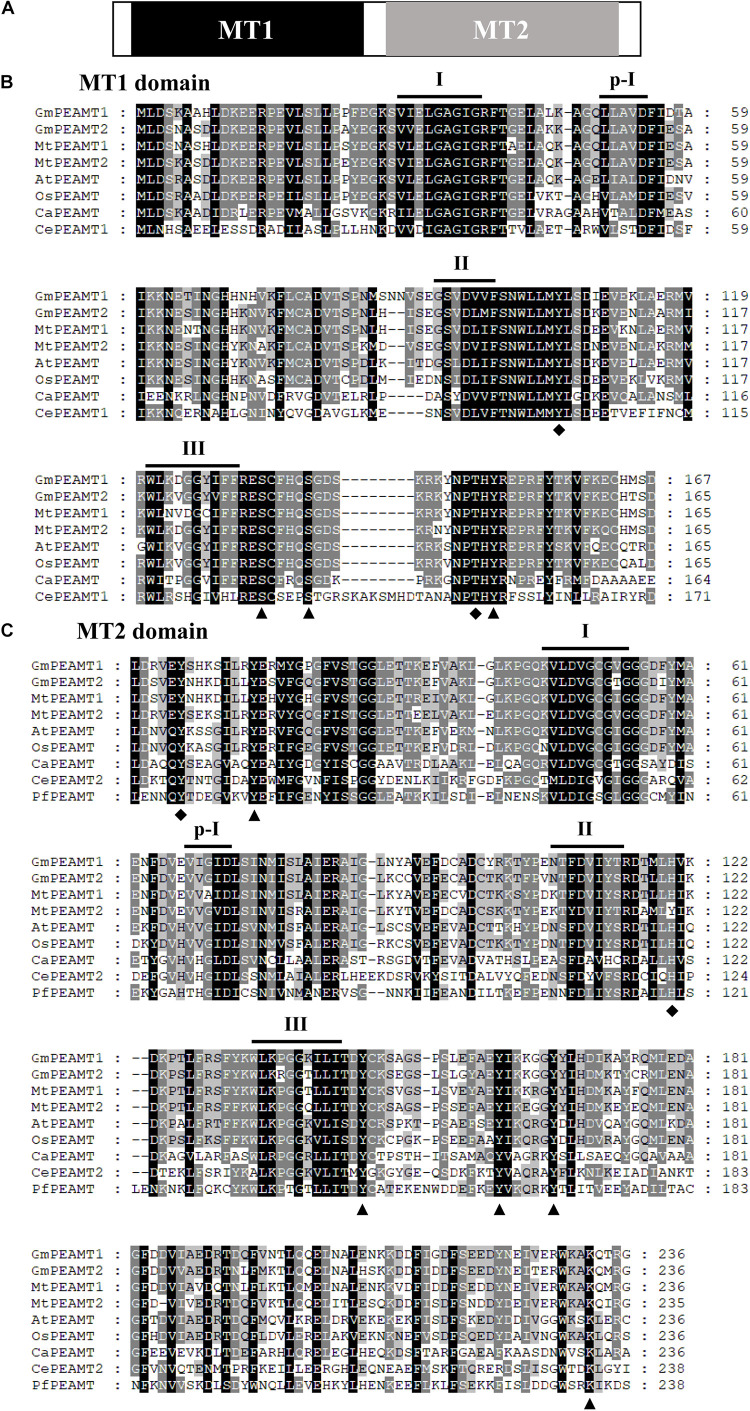
Multiple amino acid sequence alignments of the two domains of PEAMTs. **(A)** A schematic diagram of plant PEAMTs. Methyltransferase domains MT1 and MT2 are shown in black and gray, respectively. **(B,C)** Multiple alignments of MT1 **(B)** and MT2 **(C)** domains. Dashes represent gaps introduced to improve the alignment. Identical and similar amino acids are shown in black and gray, respectively. The putative phosphorylation sites and catalytic sites deduced from the structural studies (Lee and Jez, 2014) are indicated by triangles and diamonds, respectively. The four SAM-binding motifs (I, p-I, II, and III) are indicated. The alignments of MT1 and MT2 domains were conducted with ClustalX 2.0 and viewed with GeneDOC. GmPEAMT1, *Glycine max* PEAMT1 (NP_001348858.1); GmPEAMT2, *Glycine max* PEAMT2 (XP_025984992); MtPEAMT1, *Medicago truncatula* PEAMT1 (XP_003619836.1); MtPEAMT2, *Medicago truncatula* PEAMT2 (XP_003631124); AtPEAMT, *Arabidopsis thaliana* PEAMT (XP_003631124); OsPEAMT, *Oryza sativa* PEAMT (XP_015622327.1); CaPEAMT, *Chlamydomonas applanata* PEAMT (LC228965-1); CePEAMT1, *Caenorhabditis elegans* PEAMT1 (NP_494991.1); CePEAMT2, *Caenorhabditis elegans* PEAMT2 (NP_504248.1); PfaPEAMT, *Plasmodium falciparum* PEAMT (XP_001350151.1).

Through filtering and domain analysis, two candidate genes named “*GmPEAMT1*” (*Glyma05g33790*) and “*GmPEAMT2*” (*Glyma07g11580*) were determined. Both *GmPEAMT1* and *GmPEAMT2* contain 11 exons and 10 introns (Supplementary Figure 1). *GmPEAMT1* encodes a predicted protein of 488 amino acids with a calculated molecular mass of 56.01 kDa, and *GmPEAMT2* encodes a predicted protein of 531 amino acids with a calculated molecular mass of 60.74 kDa. Their isoelectric points are both less than 7. GmPEAMT1 and GmPEAMT2 contain a methyltransferase (MT) domain at the N-terminal and the C-terminal, respectively (Figure 1A). Each domain comprises four SAM-binding motifs (I, p-I, II, and III). It is found that the arrangement of motif I in GmPEAMT1 and GmPEAMT2 meets the standard requirement proposed by Clawson (Clawson et al., 1990). The alignment analysis shows that both MT1 and MT2 domains of GmPEAMTs are homologous to the respective domains in the PEAMTs of plants, nematodes, and *plasmodia*. Most phosphorylation sites and catalytic sites found in nematodes and plasmodium PEAMT (Lee and Jez, 2014) are conserved in soybean (Figures 1B,C). Neither GmPEAMT1 nor GmPEAMT2 has a transmembrane domain (Supplementary Figure 2).

**FIGURE 2:**
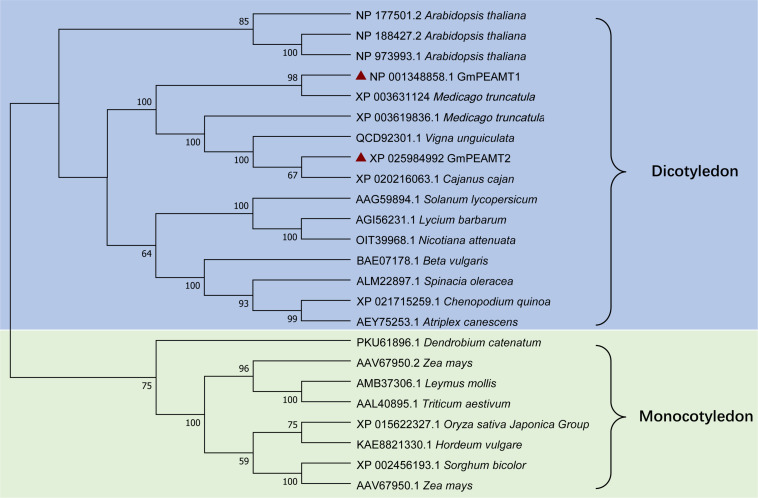
Phylogenetic analyses of full-length plant PEAMTs. Phylogenetic analyses were conducted in MEGA X. The evolutionary history was inferred, using the neighbor-joining method. The bootstrap consensus tree inferred from 1,000 replicates is taken to represent the evolutionary history of the taxa analyzed. The percentage of replicate trees in which the associated taxa clustered together in the bootstrap test (1,000 replicates) is shown next to the branches. The evolutionary distances were computed, using the Poisson correction method. This analysis involved 24 amino acid sequences of different plant PEAMTs. Groups and subgroups identified are indicated. GmPEAMT1 and GmPEAMT2 are highlighted by red triangles.

To explore the phylogenetic relationship of GmPEAMT1 and GmPEAMT2 with other plant PEAMTs, we used the neighbor-joining method of MEGA to construct a phylogenetic tree of plant PEAMTs (Figure 2). These plant PEAMTs are separated into two clades, monocotyledon, and dicotyledon. Both GmPEAMT1 and GmPEAMT2 are clustered together with other legumes. GmPEAMT1 is closely related to *Medicago truncatula* MtPEAMT2, and GmPEAMT2 shows high similarity with *Cajanus cajan* CcPEAMT, suggesting that they may have similar functions.

### Identification of *GmPEAMT1* and *GmPEAMT2* Promoters

**FIGURE 3:**
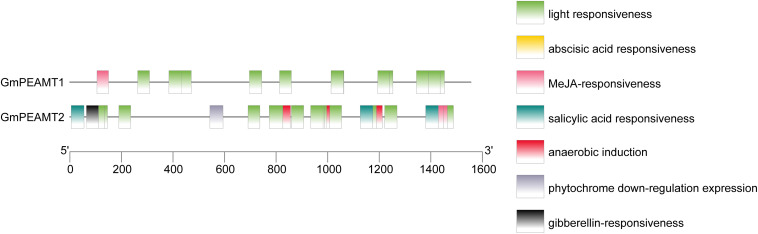
Distribution of *cis-*acting elements of *GmPEAMT* gene promoters. Different colors and shapes represent different *cis-*acting elements of *GmPEAMT* gene promoters.

The putative promoters of *GmPEAMT1* and *GmPEAMT2*, which mainly cover different classes of regulatory motifs, were isolated and sequenced. The possible *cis-*acting elements in the promoters of *GmPEAMT1* and *GmPEAMT2* were predicted by PlantCARE (Figure 3). Light response elements are found to be the most abundant *cis-*acting elements in the regions of *GmPEAMT1* and *GmPEAMT2* promoters. In addition, there is a methyl jasmonate response element in the promoter region of *GmPEAMT1*. Many plant hormone response elements are also distributed in the promoter region of *GmPEAMT2*, such as abscisic acid response elements, salicylic acid response elements, and gibberellin response elements.

### Tissue Expression Analysis of *GmPEAMT1* and *GmPEAMT2*

**FIGURE 4:**
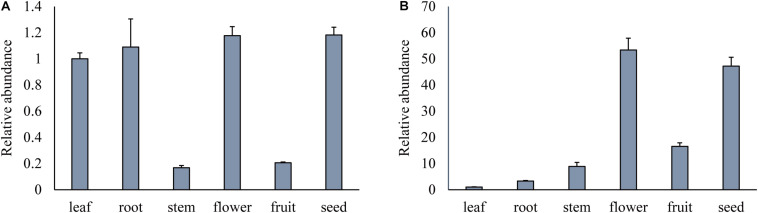
Relative expression of *GmPEAMTs* in different tissues. Relative transcript abundance of *GmPEAMT1* **(A)** and *GmPEAMT2* **(B)** in vegetative and reproductive organs was quantified by qRT-PCR and normalized to the abundance of *GmTUBLIN*. Expression analyses were relative to the expression amounts of the *GmPEAMT1* and *GmPEAMT2* in the leaf sample. The error bars represent the standard error of three replicates. Each replicate corresponded to the three individual plants.

To examine the expression patterns of *GmPEAMT1* and *GmPEAMT2*, the relative expression level of *GmPEAMTs* in different tissues during the vegetative and reproductive stages was obtained by qRT-PCR (Figure 4). Transcripts of both *GmPEAMT1* and *GmPEAMT2* can be detected in all tissues, but their expression patterns are different. *GmPEAMT1* has high expression levels in roots, leaves, flowers, and seeds, but low expression levels in stems and fruits. *GmPEAMT2* is most abundant in flowers and seeds, but hardly expressed in roots, stems, leaves, and fruits. The gene expression profile indicates that GmPEAMT1 may play important roles throughout the soybean growth period, while GmPEAMT2 is essential for flower and seed development.

### Subcellular Localization of GmPEAMT1 and GmPEAMT2

**FIGURE 5:**
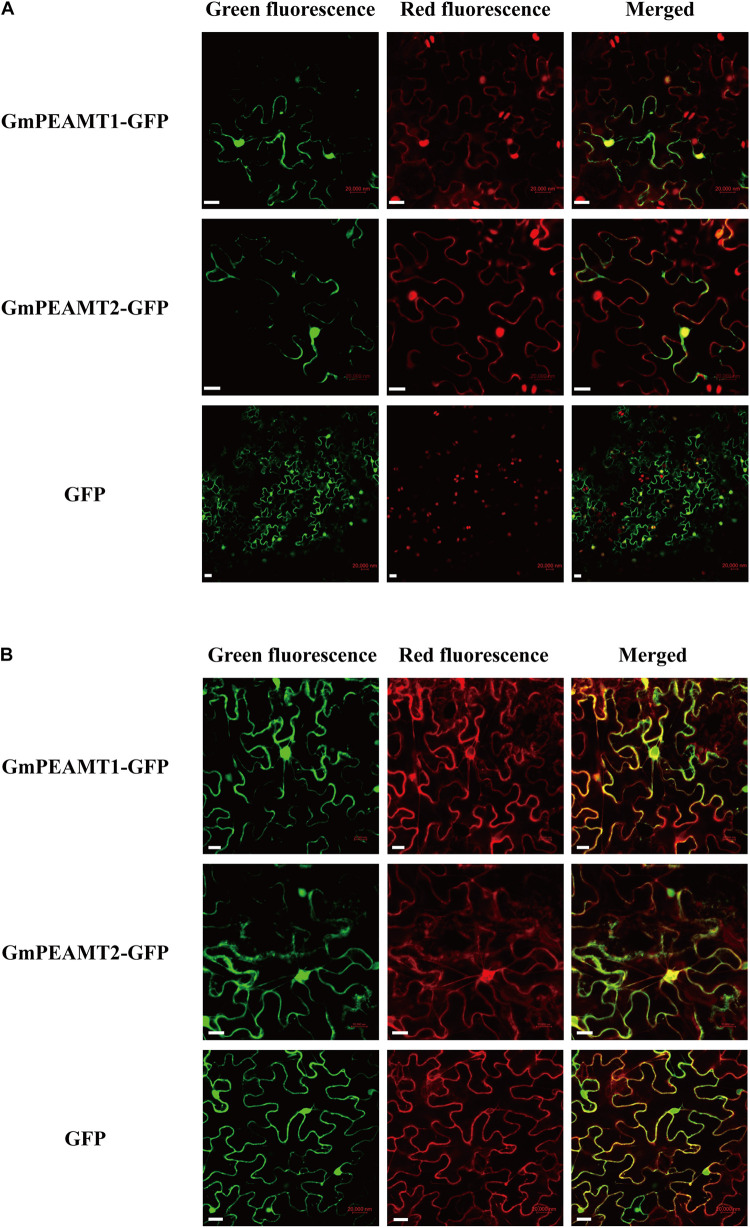
Subcellular localization of GmPEAMT proteins. The GmPEAMT–GFP fusion proteins were co-expressed with the nucleus marker RFP **(A)** and the endoplasmic reticulum marker RFP **(B)** in *N. benthamiana*, respectively. The green fluorescent, red fluorescent, and merged images are shown. The free GFPs driven by CaMV 35S were used as controls. Scale bars = 20 μm.

To determine the proteins localization in plant cells, the GmPEAMT-GFP fusion expression vector was successfully transformed into *N. benthamiana* leaves by *Agrobacterium tumefaciens*. The green fluorescent signals of GmPEAMT1-GFP fusion proteins partly coincided with the red fluorescent signals of nucleus marker proteins (Figure 5A). Notably, expression of GmPEAMT1-GFP led to the formation of punctate fluorescent signals with a radial network, particularly around the signals of nucleus marker proteins. Further research found that the radial network signals of GmPEAMT1-GFP overlapped with the red fluorescent signals of the endoplasmic reticulum marker proteins (Figure 5B). The location of GmPEAMT2 was consistent with GmPEAMT1 (Figures 5A,B). The signals of the empty plasmid pFGC5941-GFP were distributed throughout the whole cell (Figures 5A,B). The results reveal that GmPEAMT1 and GmPEAMT2 localize in the nucleus and endoplasmic reticulum.

### Functional Analysis of GmPEAMT1 and GmPEAMT2 in Yeast

**FIGURE 6:**
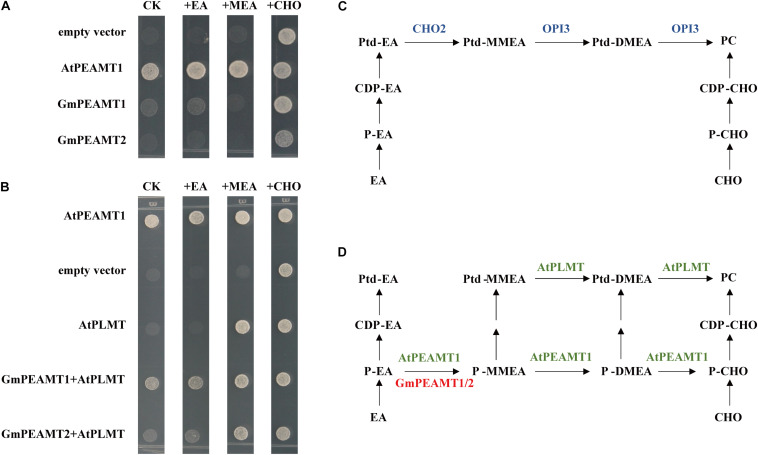
Functional complementation of GmPEAMTs in *Saccharomyces cerevisiae*. **(A,B)** Heterologous complementation of PCho-dependent growth of the *S. cerevisiae cho2 opi3* mutant. Yeast mutant strain *cho2 opi3* harboring empty vectors (negative controls), Arabidopsis *PEAMT1* (positive controls), *GmPEAMT1*, *GmPEAMT2*, *AtPLMT*, *GmPEAMT1*+*AtPLMT*, and *GmPEAMT2*+*AtPLMT* were grown on agar supplemented with minimal media and 2% (w/v) galactose, and 0-mM ethanolamine (CK) or 1-mM ethanolamine (EA) or 1-mM monomethylethanolamine (MEA) or 1-mM choline (CHO). These transformants were grown at 37°C for 3 days. Different transformants and additives are indicated at the left and the top of the images, respectively **(C,D)**. The currently proposed PC biosynthesis pathway in *S. cerevisiae* and plants, respectively (Keogh et al., 2009; Liu et al., 2018). The methyltransferases involved in the synthesis of PC in *S. cerevisiae*, Arabidopsis, and soybeans are shown in blue, green, and red. Ptd-EA, phosphatidylethanolamine; Ptd-MMEA, phosphatidylmonomethylethanolamine; Ptd-DMEA, phosphatidyldimethylethanolamine; PC, phosphatidylcholine; CDP-EA, cytidine diphosphate-ethanolamine; CDP-CHO, cytidine diphosphate-choline; P-EA, ethanolamine-phosphate; P-MMEA, monomethylethanolamine-phosphate; P-DMEA, dimethylethanolamine-phosphate; P-CHO, phosphate-choline; EA, ethanolamine; Cho, choline; PEAMT, phosphoethanolamine methyltransferase; and PLMT, phospholipid methyltransferase.

Yeast cells lack PEAMT activity, and the synthesis of PC *in vivo* relies on PLMT1/CHO2 and PLMT2/OPI3 to catalyze the three-step methylation of Ptd-EA to PC (Figure 6C). The deletion of *CHO2* and *OPI3* in yeast will completely hinder the biosynthesis of PC, resulting in a temperature-sensitive growth defect at 37°C, which is rescued by exogenous supplementation of choline (Kodaki and Yamashita, 1987, 1989; Keogh et al., 2009). To examine whether GmPEAMT1 and GmPEAMT2 encode a functional methyltransferase to produce P-Cho, we took advantage of the *cho2 opi3* mutant and observed its growth when expressing *GmPEAMT1* or *GmPEAMT2* under the control of GAL1 inducible promoters. The yeast *cho2 opi3* mutant transformed into empty vectors was used as a negative control, and the yeast *cho2 opi3* mutant transformed into *AtPEAMT1* was used as a positive control, as *AtPEAMT1* expression could fully rescue *cho2 opi3* mutant growth in the absence of exogenous choline (Figure 6D; Bolognese and McGraw, 2000). As shown in Figure 6A, the yeast *cho2 opi3* mutant expressing GmPEAMT1 or GmPEAMT2 alone could not restore normal growth in the absence of exogenous choline. These results suggest that GmPEAMT1 and GmPEAMT2 cannot continuously catalyze the three-step methylation of P-EA to P-Cho. At least one of the three steps has defects.

To further determine the catalytic abilities of GmPEAMT at the phosphate level, we co-expressed *AtPLMT* and *GmPEAMT* in the yeast *cho2 opi3* mutant. AtPLMT has a similar function to OPI3, which can catalyze the two-step methylation from Ptd-MMEA to PC *via* Ptd-DMEA (Figure 6D; Keogh et al., 2009). As shown in Figure 6B, the yeast *cho2 opi3* mutant co-expressing GmPEAMT1 and AtPLMT could restore normal growth without exogenous additives. In addition, co-expressing of GmPEAMT2 and AtPLMT could partly restore the growth of *cho2 opi3* cells. These results indicate that GmPEAMT1 and GmPEAMT2 can at least catalyze the methylation from P-EA to P-MMEA (Figure 6D).

### Functional Verification of GmPEAMT1 and GmPEAMT2 in Arabidopsis

**FIGURE 7:**
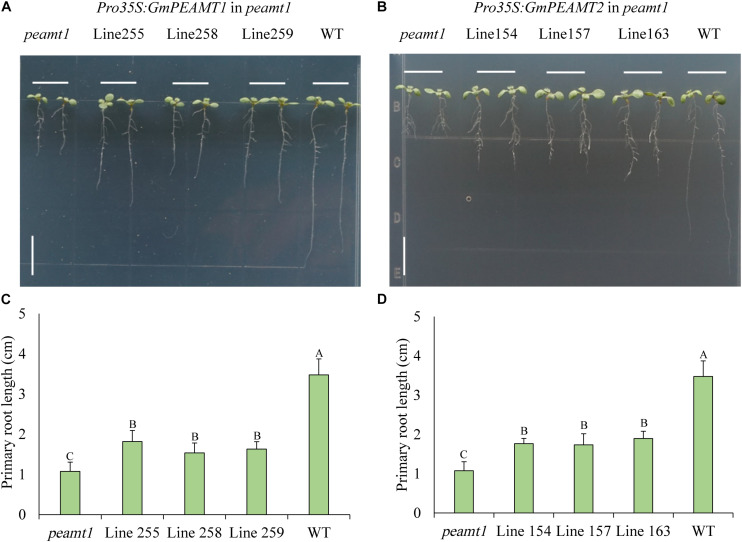
Functional verification of GmPEAMTs in *Arabidopsis thaliana* **(A,B)**. The representative phenotype of 8-day-old seedlings, from left to right: *peamt1* mutant, three independent transgenic lines expressing *Pro35S:* Gm*PEAMT1* (lines 255, 258, and 259) or *Pro35S: GmPEAMT2* (lines 154, 157, and 163) in the *peamt1* background and WT (Col-0). Seedlings were grown along vertical plates on 1% agar, supplemented with ½ MS. Scale bars = 1 cm **(C,D)**. The primary root lengths of 8-day-old Arabidopsis seedlings. The data are shown as means ± standard deviation (*n* = 10). The statistical significance was evaluated at the 0.01 probability level by Duncan’s multiple range test and indicated by different uppercase letters.

To further investigate the biochemical functions of GmPEAMT1 and GmPEAMT2 *in planta*, we individually expressed *GmPEAMT1* and *GmPEAMT2* in the Arabidopsis *peamt1* mutant, which exhibits a distinctive short-root phenotype (Cruz-Ramírez et al., 2004). As shown in Figure 7A, *peamt1* roots were significantly shorter than wild-type roots, as expected. The root length of the *peamt1* mutant expressing *GmPEAMT1* is longer significantly than *peamt1* (Figure 7C), indicating that the heterologous expression of *GmPEAMT1* in the *peamt1* mutant can partially restore normal root development but cannot revert to the wild-type level. The *peamt1* mutants expressing *GmPEAMT2* have a similar phenotype to the *peamt1* mutants, expressing *GmPEAMT1* (Figures 7B,D). These results demonstrate that GmPEAMT1 and GmPEAMT2 are partly homologous to AtPEAMT1 *in planta*, and there is some functional redundancy between GmPEAMT1 and GmPEAMT2.

## Discussion

### Possible Functional Differences Between GmPEAMTs and Other Plants PEAMTs

Phosphatidylcholine is an essential component of plant cell membranes (Meijer and Munnik, 2003). The PC synthesis pathways of different plants are distinct (Datko and Mudd, 1988b; Williams and Harwood, 1994; Lykidis, 2007). This difference is mainly reflected in the methylation at the level of phosphate and phosphatidyl mediated by PEAMT and PLMT, respectively (Keogh et al., 2009; Chen et al., 2018a). In this study, the coding sequences and promoters of *PEAMTs* were successfully isolated from soybeans. GmPEAMT1 and GmPEAMT2 are very similar to PEAMTs of other plants, such as Arabidopsis (Bolognese and McGraw, 2000) and spinach (Nuccio et al., 2000) in terms of protein size and an isoelectric point. Protein domain analysis shows that the MT1 and MT2 domain sequences of plant PEAMT are highly conserved, especially the catalytic residues and phosphorylation sites, which found in some nematodes or plasmodia (Lee and Jez, 2014), are also present in GmPEAMT1 and GmPEAMT2, indicating that GmPEAMT1 and GmPEAMT2 may have similar biological functions to other plant PEAMTs.

GmPEAMT1 and GmPEAMT2 were predicted to localize in the cytoplasm based on the protein properties, such as hydrophilicity, the lack of transmembrane structure, and the lack of organelle-targeting signal sequences. The reported plant PEAMTs are all located in the cytoplasm (Bolognese and McGraw, 2000; Smith et al., 2000). However, the results of subcellular localization show that GmPEAMT1 and GmPEAMT2 are located in the nucleus and endoplasmic reticulum, which indicates that GmPEAMT1 and GmPEAMT2 may have different properties from other plant PEAMTs. The phenomenon of non-unique subcellular localization is common in plants. The localization of some transporters, transcription factors or photoreceptor proteins will change with external signals. For example, it is reported that the localization of phosphate transporter PHT1 is affected by phosphorus content. When phosphorus is sufficient, PHT1 is phosphorylated and localized in the endoplasmic reticulum, while, when phosphorus is deficient, PHT1 is dephosphorylated and carried to the cell membrane by PHF1 (Ham et al., 2018). Therefore, the non-uniqueness of GmPEAMTs position may have a certain relationship with its response to external signals, which requires further experimental verification.

### The Potential of *GmPEAMT1* and *GmPEAMT2* to Participate in Plant Stress Response

When responding to various abiotic stresses, plants will induce the expression of specific genes through their signal transduction mechanisms, thereby reducing the harm of stress to plants, and this induced expression is related closely to the *cis-*acting elements in the upstream promoter region of the gene and the corresponding transcription factors (Liu et al., 2014). The prediction of the *cis-*acting elements in the promoters of *GmPEAMT1* and *GmPEAMT2* shows that a large number of light response elements, some plant hormone response elements, and stress response elements are distributed in the promoter region. The promoters of corn (Gou, 2017), *Suaeda liaotungensis* (Xie, 2013), and *Salicornia* (Ma, 2008) PEAMTs also contain these elements. PEAMTs of many dicotyledonous plants have light-responsive characteristics. For example, when measuring the phosphoethanolamine methyltransferase activity of spinach, it was found that its activity was highest at the end of the photoperiod and lowest at the end of the dark period. When the plant was exposed to the light again, the activity would be restored (Weretilnyk et al., 1995). Nuccio found that salt treatment improved the expression level of *PEAMT* in spinach, and then increased the production of choline to promote the accumulation of betaine (Nuccio et al., 2000). Low-temperature treatment and salt treatment will lead to the upregulation of wheat *PEAMT* and corresponding protein expression. The corresponding increase in enzyme activity may be related to the accumulation of betaine in wheat (Charron et al., 2002). These findings are consistent with the results in this study. It is preliminarily inferred that GmPEAMT1 and GmPEAMT2 are both plant stress-related genes, and their promoters may have the characteristics of responding to abiotic stress.

### The Specificity of GmPEAMT1 and GmPEAMT2 Function

Through the application of yeast heterologous complementation, some plant PEAMTs have been biochemically verified, such as spinach (Nuccio et al., 2000) and Arabidopsis (Bolognese and McGraw, 2000; Chen et al., 2018a,b). Deletion of *CHO2* and *OPI3* involved in the methylation reaction of PC biosynthesis at the phosphatidyl level is lethal unless choline is exogenously provided (Kodaki and Yamashita, 1987; Summers et al., 1988; Preitschopf et al., 1993; Keogh et al., 2009). AtPLMT is homologous to OPI3, which can catalyze the transformation of Ptd-MMEA→Ptd-DMEA→PC (Keogh et al., 2009). The independent expression of GmPEAMT1 or GmPEAMT2 in the yeast *cho2 opi3* mutant cannot compensate for its growth defect. In the absence of exogenous choline, co-expression of AtPLMT and GmPEAMT1 or GmPEAMT2 enabled the *cho2 opi3* mutant to grow. These results indicate that GmPEAMTs can at least catalyze the first step of methylation at the phosphate level to convert P-EA to P-MMEA. Then P-MMEA is converted into Ptd-MMEA through the nucleotide pathway, and finally, PC is formed under the catalysis of AtPLMT in *cho2 opi3* cells. In soybeans, it has been proved that only the first step methylation (P-EA→P-MMEA) conducts at the phosphate level by the metabolic analysis (Datko and Mudd, 1988a). Therefore, it is deduced that GmPEAMT1 and GmPEAMT2 can only catalyze the methylation of PEA to P-MMEA.

Studies have shown that the MT1 domain of PEAMT is responsible for catalyzing the initial methylation reaction from P-EA to P-MMEA; the MT2 domain catalyzes the two-step subsequent methylation reaction from P-MMEA to P-Cho *via* P-DMEA (BeGora et al., 2010; Lee and Jez, 2013, 2014, 2017). Although GmPEAMT1 and GmPEAMT2 have the MT2 domain, they cannot catalyze the last two steps of methylation. The loss of catalytic activity may result from mutations in some key positions of the MT2 domain. However, studies have also shown that there is no absolute correspondence between structural domains and catalytic functions. For example, *Caenorhabditis elegans* PEAMT1 can only methylate P-EA to P-MMEA, while the second enzyme PEAMT2 can methylate P-MMEA and P-DMEA, but they both have two domains. However, a single protein has only one methyltransferase domain to function, and the other does not have catalytic activity (Lee and Jez, 2013). What is more, *Plasmodium falciparum* PEAMT only has one domain to catalyze a continuous three-step methylation reaction (Reynolds et al., 2008).

In animals and yeast, the three-step methylation reaction on the phosphatidyl group is the only way for these organisms to *de novo* synthesis PC, and PLMT is the key enzyme that functions in this pathway (Keogh et al., 2009). However, the phosphorylation pathway for *de novo* synthesis of PC in plants starts with the methylation of P-EA by PEAMT (McNeil et al., 2001). The subsequent two-step methylation may occur at the phosphate level or the phosphatidyl level, depending on the species (Lykidis, 2007). For example, in *Lemna* and carrot (*Daucus carota* L.), the sequential methylation of P-EA and the sequential methylation of Ptd-MMEA are carried out simultaneously (Datko and Mudd, 1988b). Olive (*Olea europaea* L.) is more extraordinary than the first two-step methylation occurs at the phosphate level, and the last step occurs at the phosphatidyl level (Williams and Harwood, 1994). These results all illustrate the considerable differences in the PC synthesis pathway between different plants and the diversity of PEAMT functions. The differences in the role and necessity status of PEAMT among plants are also worthy of our in-depth exploration.

### Possible Reasons for the Difference in the PEAMT Function Between Arabidopsis and Soybeans

In order to better understand the role of *PEAMT* in plants, we analyzed the biological function of GmPEAMT1 and GmPEAMT2 through the genetic transformation in Arabidopsis. Similar to PEAMTs in most plants, AtPEAMT1 has two methyltransferase domains, MT1 and MT2, which can catalyze the three-step methylation reaction of P-EA to P-Cho at the phosphate level (Bolognese and McGraw, 2000). When *AtPEAMT1* was mutated by a T-DNA insertion, the root growth of the mutant plants was greatly restricted (Mou et al., 2002; Cruz-Ramírez et al., 2004). There were severe defects in the meristem and elongation zone, such as the abnormal morphology of the epidermal cell. In addition, the inhibition of PC synthesis also induces the death of root epidermal cells (Cruz-Ramírez et al., 2004). In this study, *GmPEAMT1* and *GmPEAMT2* were driven by the 35S promoter and transferred into the Arabidopsis *peamt1* mutant. The overexpression of GmPEAMT1 and GmPEAMT2 in the *peamt1* mutant partially restored the root growth of the mutant, indicating that GmPEAMT1 and GmPEAMT2 are similar to AtPEAMT1, which play an essential role in maintaining the normal growth and development of the root.

The introduction of *GmPEAMT1* and *GmPEAMT2* did not restore the root length of the mutant to the level of wild type, which indicates that, in the biosynthesis of PC, GmPEAMT1, and GmPEAMT2 may have similar but not identical biochemical functions to AtPEAMT1. This may result from the difference of substrate catalytic ability between soybeans and Arabidopsis. Yeast complementation verification results show that GmPEAMT1 and GmPEAMT2 can catalyze the methylation of PEA to P-MMEA. In contrast, other plant PEAMTs represented by Arabidopsis can catalyze the triple methylation of PEA to P-Cho (Chen et al., 2018b). The difference in tissue expression patterns and the subcellular localization between *GmPEAMT1* and *GmPEAMT2* may also result in the different functions between soybeans and Arabidopsis.

It is also worth noting that the overexpression of *GmPEAMT1* and *GmPEAMT2* in the *peamt1* mutant has similar phenotypes, indicating that *GmPEAMT1* and *GmPEAMT2* have a certain degree of functional redundancy in maintaining the normal growth and development of the root. This kind of genetic redundancy is widespread in organisms. It is a coping strategy to reduce the impact of unfavorable external environmental factors on their survival, which gradually formed in the evolution process of organisms adapting to the environment. Through the genetic redundancy, phenotypic changes caused by a knockout or loss of function of a gene can be reduced (Li et al., 2018). Given the difference in tissue expression patterns of GmPEAMT1 and GmPEAMT2, whether they have different functions in other processes of plant growth and development remains to be explored. Elucidating the synergistic but unequal functions of GmPEAMT1 and GmPEAMT2 in soybean PC synthesis, and the contribution of GmPEAMT-mediated phosphate level methylation to PC synthesis will be important future effort.

In summary, the identification and functional analysis of PEAMTs in soybeans enhanced our understanding of the synthesis pathway of phosphatidylcholine in legumes. It provides valuable materials for further exploration of the specific pathways of lipid synthesis between different plants and the massive variability in the regulation of this process. At the same time, it is also of great significance for improving stress resistance and soybean quality.

## Data Availability Statement

The datasets presented in this study can be found in online repositories. The names of the repository/repositories and accession number(s) can be found in the article/Supplementary Material.

## Author Contributions

YC and YS conceived the study. XW contributed to the cultivation of test materials. WC performed the qRT-PCR. QY performed the subcellular localization experiment. XJ carried out the functional verification experiments in Arabidopsis and yeast, analyzed the data, and drafted the manuscript. All authors contributed to the article and approved the submitted version.

## Conflict of Interest

The authors declare that the research was conducted in the absence of any commercial or financial relationships that could be construed as a potential conflict of interest.

## Publisher’s Note

All claims expressed in this article are solely those of the authors and do not necessarily represent those of their affiliated organizations, or those of the publisher, the editors and the reviewers. Any product that may be evaluated in this article, or claim that may be made by its manufacturer, is not guaranteed or endorsed by the publisher.

## Footnotes

1
http://www.arabidopsis.org/abrc/

2
https://phytozome.jgi.doe.gov

3
http://smart.embl-heidelberg.de/

4
http://gsds.cbi.pku.edu.cn/

5
http://www.cbs.dtu.dk/services/TMHMM/
