# Supplementary material for: Cloning and Functional Identification of Phosphoethanolamine Methyltransferase in Soybean (Glycine max)
Source: Front Plant Sci. 2021 Jul 27;12:612158. doi: 10.3389/fpls.2021.612158 (PMC8353235; doi:10.3389/fpls.2021.612158)
Supplement: Supplementary file 1 [file Table_1.DOCX]

Supplementary Material

# Supplementary Tables

**Supplementary Table 1.** Primers used in this study

| Primer name | Sequence (5’-3’) | Usage description |
| --- | --- | --- |
| GmPEAMT1-CLO-F | ATGGATGAGCGTCACATT | Gene amplification |
| GmPEAMT1-CLO-R | TCAATTTTTCTTGGCAAT |  |
| GmPEAMT2-CLO-F | ATGGAGGTAAGAACGAAC |  |
| GmPEAMT2-CLO-R | TTATTTCTTGGCAATGAA |  |
| GmPEAMT1-PRO-F | CTAGCAGCTGAGGGACATAATCC | Promoter amplification |
| GmPEAMT1-PRO-R | AGCTGAAAGCGGTGGGTGT |  |
| GmPEAMT2-PRO-F | gacaccttagaaattctcacatgt |  |
| GmPEAMT2-PRO-R | TTATAGCTTTCCCGTCATTTTTTTC |  |
| GmTUBULIN-RTP-F | GGAGTTCACAGAGGCAGAG | Real-time fluorescence quantitative PCR |
| GmTUBULIN-RTP-R | CACTTACGCATCACATAGCA |  |
| GmPEAMT1-RTP-F | GCCGCTCATCTCGACAAAGA |  |
| GmPEAMT1-RTP-R | ATTGACCAGCTTTCAGAGCCA |  |
| GmPEAMT2-RTP-F | GCGTGGAGGTACACTTCTGA |  |
| GmPEAMT2-RTP-R | AGCATTCTCGAGCATCCGAC |  |
| GmPEAMT1-GFP-F | agcgagctcATGGATGAGCGTCACATT | Construction of fusion expression vector |
| GmPEAMT1-GFP-R | agcccatggTATTTTTCTTGGCAATGAA |  |
| GmPEAMT2-GFP-F | agccccgggATGGAGGTAAGAACGAAC |  |
| GmPEAMT2-GFP-R | agcccatggTTTTCTTGGCAATGAACAA |  |
| AtPLMT-YEA-F | agcggtaccATGGGATTATTGGCCG | Construction of yeast expression vector |
| AtPLMT-YEA-R | agctctagaTCATGAGATGGATTTGGC |  |
| AtPEAMT-YEA-F | AGGAGAAAAAACCCCGGATCCATGGCTGCATCGTACG |  |
| AtPEAMT-YEA-R | CAACTTCTGTTCCATGTCGACATTCTTGTTGGCGATG |  |
| GmPEAMT1-YEA-F | AGGAGAAAAAACCCCggatccATGGATGAGCGTCACATT |  |
| GmPEAMT1-YEA-R | CAACTTCTGTTCCATgtcgacATTTTTCTTGGCAATGAA |  |
| GmPEAMT2-YEA-F | AGGAGAAAAAACCCCggatccATGGAGGTAAGAACGAAC |  |
| GmPEAMT2-YEA-R | CAACTTCTGTTCCATgtcgacTTTCTTGGCAATGAACAA |  |
| GmPEAMT1-PLA-F | agcccATGGATGAGCGTCACATT | Construction of plant expression vector |
| GmPEAMT1-PLA-R | agctctagaTCAATTTTTCTTGGCAAT |  |
| GmPEAMT2-PLA-F | agcccatggATGGAGGTAAGAACGAAC |  |
| GmPEAMT2-PLA-R | agcggatccTTATTTCTTGGCAATGAA |  |
