# Supplementary material for: Cloning and Functional Identification of Phosphoethanolamine Methyltransferase in Soybean (Glycine max)
Source: Front Plant Sci. 2021 Jul 27;12:612158. doi: 10.3389/fpls.2021.612158 (PMC8353235; doi:10.3389/fpls.2021.612158)
Supplement: Supplementary file 2 [file Data_Sheet_1.PDF]

Supplementary Material

1 Supplementary Figures

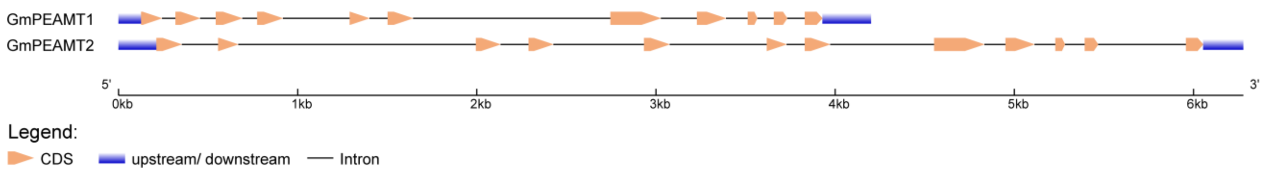

Supplementary Figure 1. Intron-exon structure of *GmPEAMT*. Schematic representation of *GmPEAMT1* and *GmPEAMT2* genome structure were built by online program GSDS. Orange triangles indicate CDS, blue rectangles indicate UTRs, and black line indicate intron.
