# Supplementary material for: Cloning and Functional Identification of Phosphoethanolamine Methyltransferase in Soybean (Glycine max)
Source: Front Plant Sci. 2021 Jul 27;12:612158. doi: 10.3389/fpls.2021.612158 (PMC8353235; doi:10.3389/fpls.2021.612158)
Supplement: Supplementary file 3 [file Data_Sheet_2.PDF]

Supplementary Material

1 Supplementary Figures

A

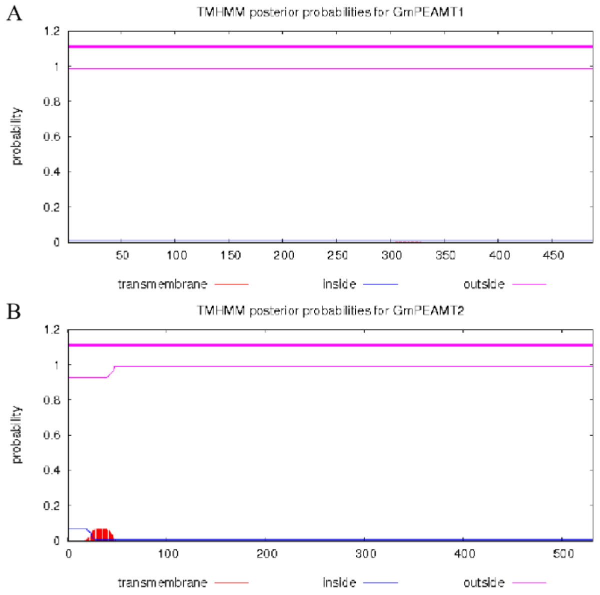

Supplementary Figure 2. Prediction of transmembrane helices in GmPEAMT. **A**: GmPEAMT1; **B**: GmPEAMT2. Prediction of TM helices in proteins were performed using the TMHMM methodbased on a hidden Markove model (http://www.cbs.dtu.dk/services/TMHMM-2.0/).
